# BMP-2 Induces Versican and Hyaluronan That Contribute to Post-EMT AV Cushion Cell Migration

**DOI:** 10.1371/journal.pone.0077593

**Published:** 2013-10-11

**Authors:** Kei Inai, Jessica L. Burnside, Stanley Hoffman, Bryan P. Toole, Yukiko Sugi

**Affiliations:** Department of Regenerative Medicine and Cell Biology and Cardiovascular Developmental Biology Center, Medical University of South Carolina, Charleston, South Carolina, United States of America; Heart Science Centre, Imperial College London, United Kingdom

## Abstract

Distal outgrowth and maturation of mesenchymalized endocardial cushions are critical morphogenetic events during post-EMT atrioventricular (AV) valvuloseptal morphogenesis. We explored the role of BMP-2 in the regulation of valvulogenic extracellular matrix (ECM) components, versican and hyaluronan (HA), and cell migration during post-EMT AV cushion distal outgrowth/expansion. We observed intense staining of versican and HA in AV cushion mesenchyme from the early cushion expansion stage, Hamburger and Hamilton (HH) stage-17 to the cushion maturation stage, HH stage-29 in the chick. Based on this expression pattern we examined the role of BMP-2 in regulating versican and HA using 3D AV cushion mesenchymal cell (CMC) aggregate cultures on hydrated collagen gels. BMP-2 induced versican expression and HA deposition as well as mRNA expression of *versican* and *Has2* by CMCs in a dose dependent manner. Noggin, an antagonist of BMP, abolished BMP-2-induced versican and HA as well as mRNA expression of *versican* and *Has2*. We further examined whether BMP-2-promoted cell migration was associated with expression of versican and HA. BMP-2- promoted cell migration was significantly impaired by treatments with versican siRNA and HA oligomer. In conclusion, we provide evidence that BMP-2 induces expression of versican and HA by AV CMCs and that these ECM components contribute to BMP-2-induced CMC migration, indicating critical roles for BMP-2 in distal outgrowth/expansion of mesenchymalized AV cushions.

## Introduction

Cardiac valvuloseptal morphogenesis is a key morphogenetic event during formation of the four-chambered heart. During this process, two segments of endocardium — atrioventricular (AV) and outflow tract (OT) endocardium — transform into cushion mesenchyme through an epithelial- mesenchymal transformation (EMT). Mesenchymalized endocardial cushions constitute primordia of the valves and membranous septum [1-4]. Bone morphogenetic protein (BMP) signaling is found critical for AV cushion formation in studies with explant cultures [5,6] and in conditional knockout (cKO) mouse experiments [7-11]. After the initial formation of AV endocardial cushions at Hamburger and Hamilton (HH) stage-15 in the chick and embryonic day (ED) 9.5 in the mouse, BMP-2 ligand and Type I BMP receptors (BMPRs) are abundantly expressed in the expanding and maturing AV cushion mesenchyme [5,12-14]. However, early lethality of the BMP-2 myocardial cKO and BMPR1A (Alk3) endocardial cKO mice at the EMT stage [7-10] prevents investigation of the roles of BMP signaling in post-EMT AV valvuloseptal morphogenesis. 

Distal outgrowth/expansion and maturation of mesenchymalized endocardial cushions are critical morphogenetic events during post-EMT AV valvuloseptal morphogenesis. At the onset of EMT, endocardial endothelial cells transform into cushion mesenchymal cells. Transformed cushion mesenchymal cells (CMCs) invade into the acellular cardiac jelly. After EMT, during distal elongation of the AV cushions CMCs continue to migrate and spread within the expanding mesenchymalized cushions. Distal outgrowth and expansion, which begin at HH stage-25/26 in the chick, largely involve i) CMC migratory behavior and ii) expression of valvulogenic molecules by CMCs, which include extracellular matrix (ECM) components [15,16]. We and others have proposed regulatory roles for BMP in CMC differentiation after EMT [14,17]; however, the role of BMPs in regulating the valvulogenic processes, i. e. CMC migration and expression of ECM components, has not been fully elucidated in post-EMT AV cushion distal outgrowth and expansion. 

Two of the prominent ECM components in AV cushions, hyaluronan (HA) and versican interact and make macromolecular complexes in developing cartilage [18,19]. Regarding cardiac development, mouse models with deficiencies in versican (hdf mouse) [20,21] and in HA synthase 2 (*Has2* KO mouse) [22] are characterized by severe cardiac defects resulting from abnormal formation of the cardiac cushion mesenchyme at the EMT stage, indicating that these two ECM components are essential for valvuloseptal morphogenesis. However, because of the early lethality of mice with *Has2* and *versican* deficiencies at the cushion-formation stage, studying the role of these ECM molecules during later valvuloseptal morphogenesis is hampered. 

Valvuloseptal morphogenesis is thought to be regulated by a coordination of growth factor signaling and ECM interactions [23,24]; however, despite the relevance, little is known about the functional relationship of BMP and ECM and their roles in CMC migration during distal outgrowth and maturation of AV cushions. In our previous work, we demonstrated that BMP-2 and the BMP signaling pathway induced AV CMC migration [14]. In the present work, we explored the expression patterns of two major ECM components, versican and HA, during AV cushion expansion and distal outgrowth and investigated the role of BMP-2 in versican and HA production. Using a well-defined 3D CMC aggregate culture on hydrated collagen gels, we provide evidence that BMP-2 induces production of HA and versican and that these ECM components contribute to BMP-2-stimulated CMC migration during post-EMT AV cushion expansion and distal outgrowth. 

## Results

### Versican and HA are intensely expressed in AV cushion mesenchyme during AV cushion expansion and maturation

**Figure 1:**
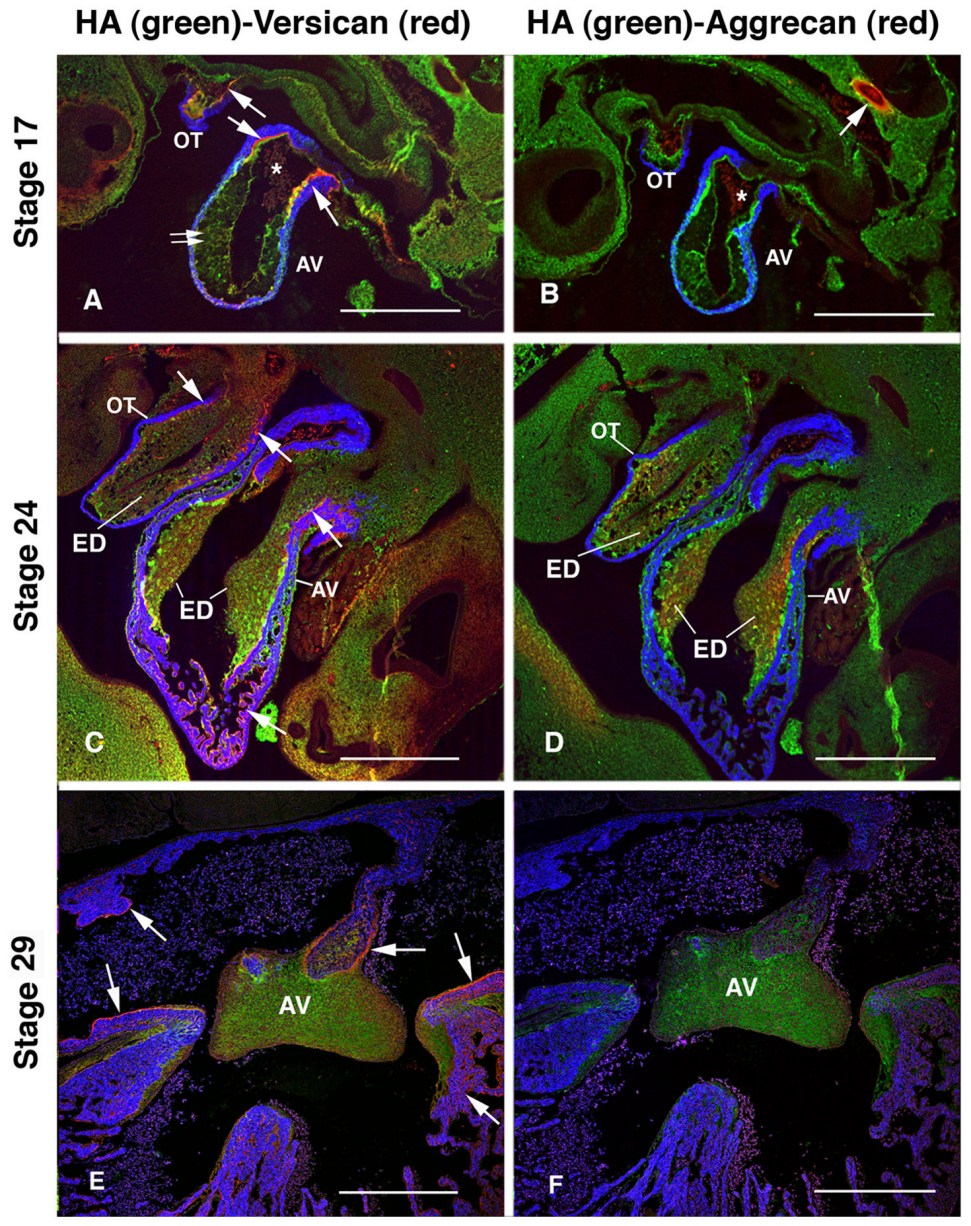
Immunohistochemical localization of hyaluronan (HA), versican and aggrecan in HH stage-17, -24 and -29 chick embryo hearts. HA deposition was detected using HA binding protein (HABP). A, C and E show HA (green) and versican (red) staining. MF20 immunostaining is shown in blue. B, D and F show HA (green) and aggrecan (red). HA is abundantly localized in outflow tract (OT) and atrioventricular (AV) cushion mesenchyme throughout early endocardial cushion forming stage (HH stage-17) to endocardial cushion maturation stage (HH stage-29). Versican expression is evident in myocardial basement membrane in the distal end of outflow (OT) and inflow tracts (arrows in A) as well as in the cushion mesenchyme (double arrows in A) at HH stage-17. Intense versican expression is seen at the myocardial-cushion mesenchyme interface (arrows in C), subendocardial space in the ventricular trabeculae (arrows in C, E) and in the OT and AV cushions (ED in C) at HH stage-24. Aggrecan expression is restricted to forming vertebra at HH stage-17 (an arrow in B). Aggrecan expression is confined to AV and OT cushions (ED in D) but not seen in the atrial and ventricular myocardium. Aggrecan expression is most prominently seen at HH stage-24 and less abundantly expressed at HH stage-29 (F). * blood in the cardiac lumen. Scale bars=50 µm.

HA, versican and aggrecan were localized in HH stage-17, -24, and -29 chick embryo hearts. HA localization was histochemically detected with hyaluronan binding protein (HABP) following the protocol described previously [25]. Production and specificity of the anti-chick versican and anti-chick aggrecan antibodies were described previously [26]. At the early stage of cardiac cushion expansion (HH stage-17), intense immunostaining of versican was observed in the basement membrane of the endocardial side of myocardium, most prominently at the distal end of the cardiac inlet and outlet (arrows in Figure 1A). HA and versican were localized in the AV cardiac jelly (double arrows in Figure 1A), whereas aggrecan expression was restricted to cartilage in the developing vertebra (an arrow in Figure 1B) but not in the myocardium. HA staining images we present in this paper do not differentiate intracellular from extracellular staining. At the middle stage of AV cushion expansion (HH stage-24), HA, versican and aggrecan were localized in the AV and OT endocardial cushions (ED in Figure 1C, 1D). In addition, intense versican expression was detected in the myocardial basement membrane most prominently in the subendocardial space of the atrial and ventricular myocardium (arrows in Figure 1C) and myocardial-cushion interface in the OT and AV cushions (arrows in Figure 1C), whereas aggrecan expression was confined to the mesenchymalized AV and OT cushions (ED in Figure 1D). We observed robust deposition of HA and versican in the AV cushion up to the cushion maturation stage at H-H stage-29 (Figure 1E, 1F). Versican expression was also prominent in the myocardial basement membrane (endocardial-myocardial interface) in the atrial myocardium and ventricular trabeculae (arrows in Figure 1E), whereas aggrecan expression was not detectible in the atrial and ventricular walls (Figure 1 F). Robust HA staining persisted in the AV cushions from the early cushion-forming stage (HH stage-17) to the cushion maturation stage (HH stage-29). Consistent with a previous report indicating declining mRNA expression of aggrecan in the heart after HH stage-20 [26], we found that aggrecan protein was most intensely expressed at HH stage-24 but was less abundantly expressed at HH stage-29 in the heart. 

### BMP-2 Treatment Increases Expression of Versican Protein and mRNA by AV Endocardial Cushion Mesenchymal Cells (CMCs)

**Figure 2:**
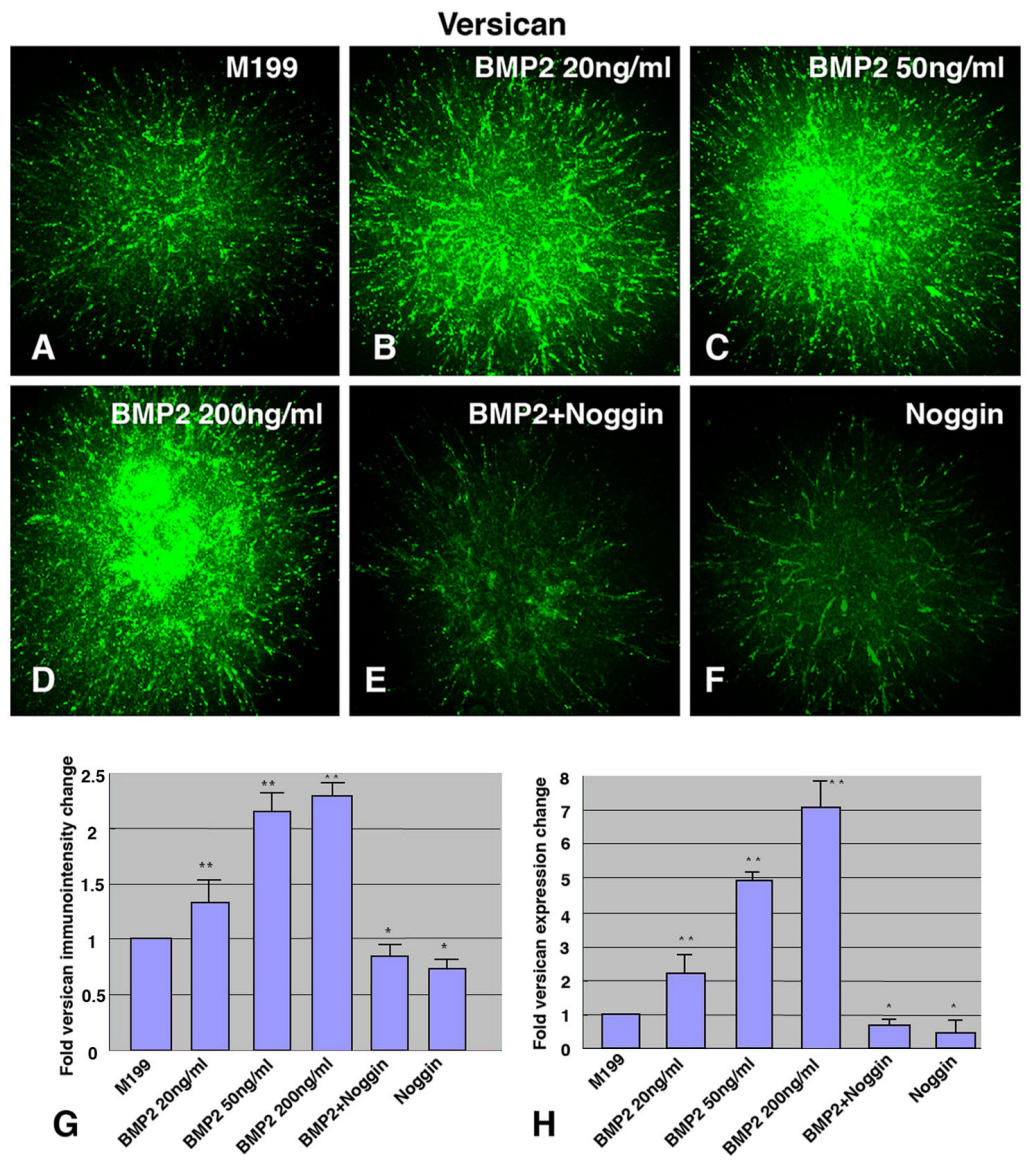
BMP-2 treatment increases versican expression by AV endocardial cushion mesenchymal cells (CMCs). (A-F) Versican immunostaining in HH stage-24 AV CMC aggregate cultures on collagen gels. The CMC aggregates were cultured in Medium 199 alone (control) (A), or with BMP-2, 20 ng/ml (B), BMP-2, 50 ng/ml (C), BMP-2, 200 ng/ml (D), BMP-2 200 ng/ml plus noggin, 500 ng/ml (E) or noggin, 500 ng/ml (F), dissolved in Medium 199. As low as 20 ng/ml of BMP-2 promoted versican immunostaining in cultured CMC aggregates. (G) Quantitative evaluation of versican immunointensity in AV CMC aggregate cultures. Immunointensity of the CMC aggregates treated with concentrations as low as 20 ng/ml of BMP-2 was significantly higher than the control (**p<0.01). Conversely, immunointensity in CMC aggregates cultured with noggin was significantly lower than the control (*p<0.05). (H) Quantitative evaluation of *versican* mRNA expression in CMC aggregate cultures. QRT-PCR data revealed an approximately 7-fold increase of *versican* expression in AV CMC aggregate cultures incubated with BMP-2 over the control cultures (**p<0.01). The potential of BMP-2 to stimulate *versican* expression was abolished when noggin was added to the cultures (*p<0.05). Vertical bars indicate ± SD of the mean. *p<0.05 ; **p<0.01 as compared to the control (M199).

Versican expression was assessed (both mRNA and protein) in AV CMC aggregates cultured on hydrated collagen gels. Versican protein expression was detected by immunostaining (Figure 2A-2F) and quantitatively evaluated by immunointensity analysis (Figure 2G) following the procedure described previously [14]. The expression of versican was significantly increased when AV CMC aggregates were cultured with concentrations as low as 20 ng/ml BMP-2 (Figure 2A-2G). Incubation with noggin, an antagonist of BMP, abolished BMP-2-induced versican expression in cultured AV CMC aggregates (Figure 2E, 2G). Incubation with noggin alone also significantly repressed endogenous versican expression in CMC aggregates when compared to the untreated control, i.e. incubation with Medium 199 alone (Figure 2A, 2F, 2G). 

We also assessed *versican* mRNA expression in AV CMC aggregates cultured on collagen gels (Figure 2H). Quantitative (Q) RT-PCR data revealed an approximately 7-fold increase of *versican* mRNA expression by AV CMC aggregates treated with BMP-2 over the untreated control (Figure 2H). As low as 20 ng/ml of exogenously added BMP-2 induced *versican* mRNA expression (Figure 2H). Incubation with noggin in the presence of exogenously added BMP-2 significantly inhibited BMP-2-stimulated expression of *versican* mRNA in AV CMC aggregates in culture. Incubation with noggin alone also significantly repressed endogenous versican mRNA expression in AV CMC aggregates cultured on the collagen gels (Figure 2H). 

### BMP-2 treatment induces HA deposition and Has2 mRNA expression by AV CMCs

**Figure 3:**
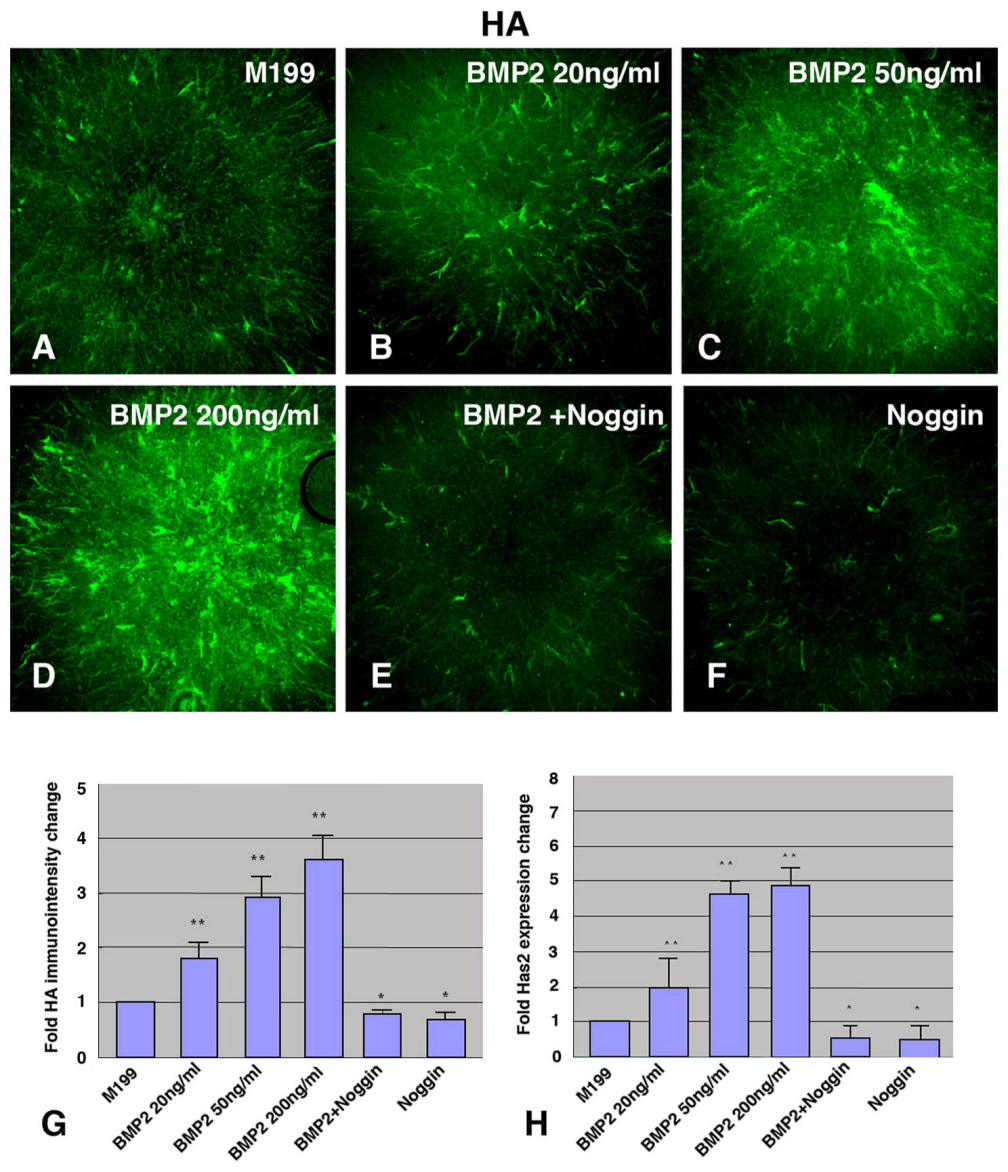
BMP-2 treatment increases HA deposition and *Has2* expression by AV endocardial cushion mesenchymal cells (CMCs). (A-F) HA staining by using hyaluronan-binding protein (HABP) in HH stage-24 AV CMC aggregate cultures on collagen gels. The CMC aggregates were cultured in Medium 199 alone (control) (A), or with BMP-2, 20ng/ml (B), BMP-2, 50ng/ml (C), BMP-2, 200 ng/ml (D), BMP-2 200 ng/ml plus noggin, 500 ng/ml (E) or noggin, 500 ng/ml (F), dissolved in Medium 199. As low as 20 ng/ml of BMP-2-stimulated HA staining in cultured CMC aggregates. (G) Quantitative evaluation of HA staining in AV CMC aggregate cultures. Staining intensity of the CMC aggregates treated with as low as 20 ng/ml of BMP-2 was significantly higher than the control (**p<0.01). Conversely, staining intensity in CMC aggregates cultured with noggin was significantly lower than the control (*p<0.05). (H) Quantitative evaluation of mRNA expression of *Has2* in CMC aggregate cultures. QRT-PCR data revealed an approximately 5-fold increase of *Has2* in CMC aggregate cultures treated with BMP-2 over control cultures (**p<0.01). BMP-2-stimulated *Has2* expression was abolished when noggin was added to the cultures (*p<0.05). Vertical bars indicate ± SD of the mean. *p<0.05; **p<0.01 as compared to the control (M199).

HA deposition was assessed with HABP histochemistry in AV CMC aggregates cultured on the hydrated collagen gels (Figure 3A-3G). HA deposition was quantitatively evaluated by stain intensity analysis (Figure 3G). Significant promotion of HA deposition was detected when AV CMC aggregates were treated with as low as 20 ng/ml BMP-2 (Figure 3A-3G). Noggin addition to the medium inhibited the potential of BMP-2 to stimulate HA deposition (Figure 3E, 3G). Incubation with noggin alone also significantly repressed endogenous HA deposition in cultured AV CMC aggregates as compared to the untreated control (Figure 3A, 3F, 3G). 

The Has’s are enzymes localized in the cell membrane that are responsible for HA production. *Has2* is the only known *Has* expressed in the embryonic heart [23]. Therefore, *Has2* mRNA expression was used as an index of HA production by CMCs in this study. *Has2* mRNA expression was assessed with QRT-PCR in AV CMC aggregates cultured on collagen gels (Figure 3H). QRT-PCR data revealed an approximately 5-fold increase of *Has2* expression by CMC aggregates treated with 50-200 ng/ml BMP-2 over the untreated controls (Figure 3H). As low as 20 ng/ml of BMP-2 induced *Has2* expression (Figure 3H). BMP-2-stimulated *Has2* mRNA expression was abolished when noggin was added to the culture. Incubation with noggin alone also significantly repressed endogenous *Has2* mRNA expression in cultured AV CMC aggregates (Figure 3H). 

### BMP-2-promoted AV cushion mesenchymal cell migration is impaired by versican siRNA treatment

**Figure 4:**
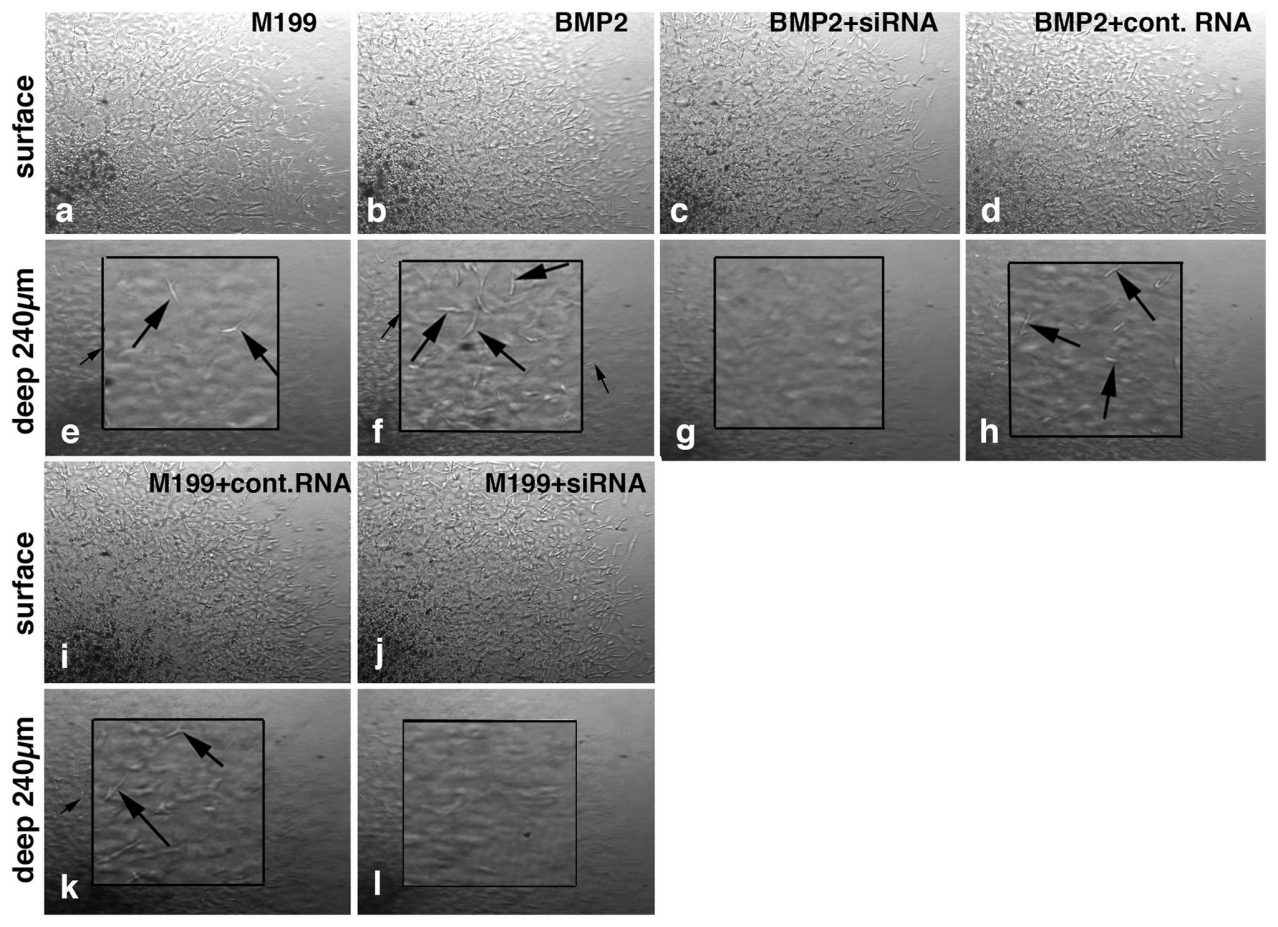
Collagen gel images of HH stage-24 AV CMC aggregate cultures treated with versican siRNA. Upper panels show the surface of the gel and the lower panels show a depth of 240 µm within the gel. Higher magnification views shown in the insets in Figures e, f, h and k indicate CMCs in the focal plane at 240µm below the surface of the gels (arrows in e, f, h and k). Note that there are no CMCs in the focal plane at 240 µm below the surface of the gels in Figures g and l. CMCs treated with BMP-2 (200 ng/ml) migrated deeper into the collagen gels (b and arrows in f) than the control cultures (M199, a and arrows in e). Versican siRNA treatment (100 nM) significantly reduced cell migration (j, l), whereas, treatments with control RNA (scrambled RNA, 100 nM) did not alter CMC migration (i and arrows in k). BMP-2-stimulated cell migration was significantly reduced when versican siRNA was added to the medium (c and g), whereas control RNA did not alter BMP-2-stimulated cell migration (d and arrows in h).

**Figure 5:**
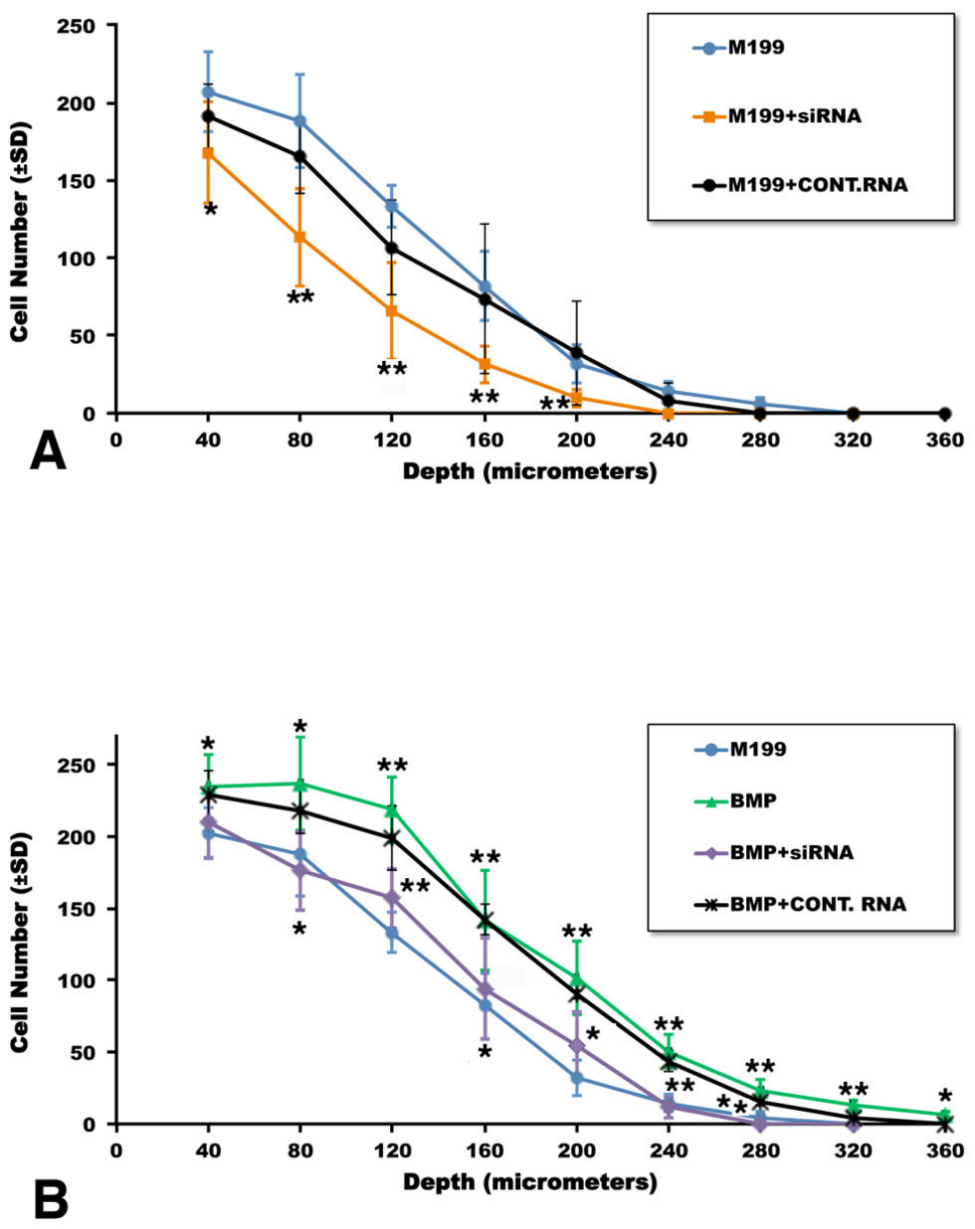
Quantitative evaluation of cell migration by HH atage-24 AV CMC aggregate cultures treated with versican siRNA. (A) CMC aggregate cultures were treated with versican siRNA (100 nM) or control (scrambled) RNA (100 nM). Versican siRNA treatment significantly reduced cell migration, whereas control (scrambled) RNA treatment did not significantly alter cell migration. *p<0.01; **p<0.001 as compared to the untreated control (M199). (B) BMP-2 (200 ng/ml)-stimulated CMC migration. BMP-2-stimulated cell migration was significantly reduced by adding versican siRNA (100 nM) to the CMC aggregates cultures, whereas control (scrambled) RNA (100 nM) did not reduce BMP-2-stimulated mesenchymal cell migration. The asterisks on the green curve indicate significant differences between BMP-2 treatment and the untreated control (M199) (blue curve). The asterisks on the purple curve indicate significant differences between treatment with BMP with BMP-2+ versican siRNA and treatment with BMP-2 (green curve). Vertical bars indicate ± SD of the mean. *p<0.01; **p<0.001. A and B represent quantitation of results for each culture condition from a minimum of three experiments, in each of which three separate aggregates were examined.

Our previous study indicates that BMP-2 induces AV CMC migration in a dose dependent manner. As low as 50 ng/ml BMP-2 promoted CMC migration into collagen gels [14]. To examine whether versican expression plays an important role in BMP-2-induced AV CMC migration, we treated CMCs with versican siRNA. Target sequences of the versican siRNA are located in the chick versican G1 domain, thus targeting all chick versican isoforms. First, the efficacy of versican siRNA treatment for versican gene silencing was verified by versican immunointensity analyses and QPCR analysis for versican mRNA expression. Versican siRNA significantly reduced versican expression, whereas control scrambled RNA did not alter versican expression by AV CMCs (Figure S1). Secondly, with versican siRNA we examined the significance of versican expression in BMP-2-promoted AV CMC migration. AV CMCs cultured with BMP-2 migrated deeper into the collagen gels (Figure 4b, 4f) than the control untreated CMCs (Figure 4a, 4e). Quantitative measurements, as shown in Figure 5B, confirmed that BMP-2-treated CMCs indeed migrated deeper (360 µm from the surface) into the gels than the control untreated CMCs (280 µm from the surface). Effects of BMP-2 were reduced when CMCs were treated with versican siRNA (100 nM) and cultured with the BMP-2-containing medium (Figure 4c, 4g). Control (scrambled) RNA did not inhibit BMP-2-induced cell migration (Figure 4d, 4h). Versican siRNA alone also repressed migration of CMCs (Figure 4j, 4l) when compared to the CMCs treated with scrambled RNA (Figure 4i, 4k). Quantitative analysis confirmed that versican siRNA treatment significantly reduced endogenous and BMP-2-promoted AV CMC migration, whereas, control (scrambled) RNA did not alter CMC migration (Figure 5A, 5B). 

### BMP-2- promoted AV cushion mesenchymal cell migration is impaired by HA oligomer treatment

**Figure 6:**
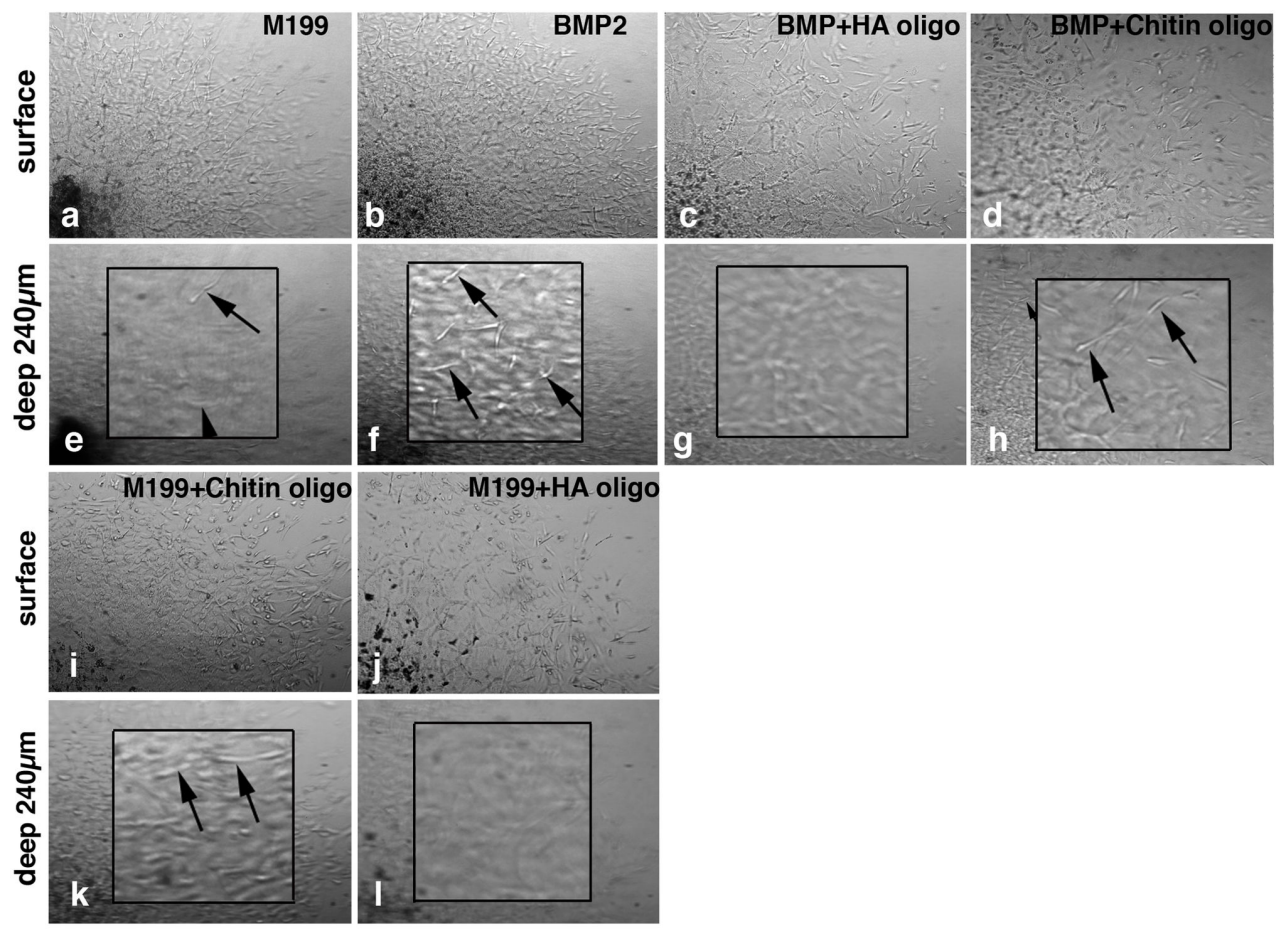
Collagen gel images of HH stage-24 AV CMC aggregate cultures treated with HA oligomers. Upper panels show the surface of the gel and the lower panels show a depth of 240µm within the gel. Higher magnification views shown in the insets in Figures e, f, h and k indicate CMCs in the focal plane at 240µm below the surface of the gels (arrows in e, f, h and k). Note that there are no mesenchymal cells in the focal plane at 240µm below the surface of the gels in Figures g and l. CMCs treated with BMP-2 (200 ng/ml) migrated deeper into the collagen gels (b and arrows in f) than the control cultures (M199, a and arrows in e). BMP-2-stimulated CMC migration was significantly reduced when HA oligomers (100 µg/ml) were added to the medium (c and g). HA oligomer treatments alone reduced mesenchymal cell migration relative to the control cultures (j and l), whereas chitin oligomers (control oligomers) (100 µg/ml) did not alter CMC migration (i, arrows in k). Chitin oligomers did not reduce BMP-2-stimulated CMC migration (d, arrows in h).

**Figure 7:**
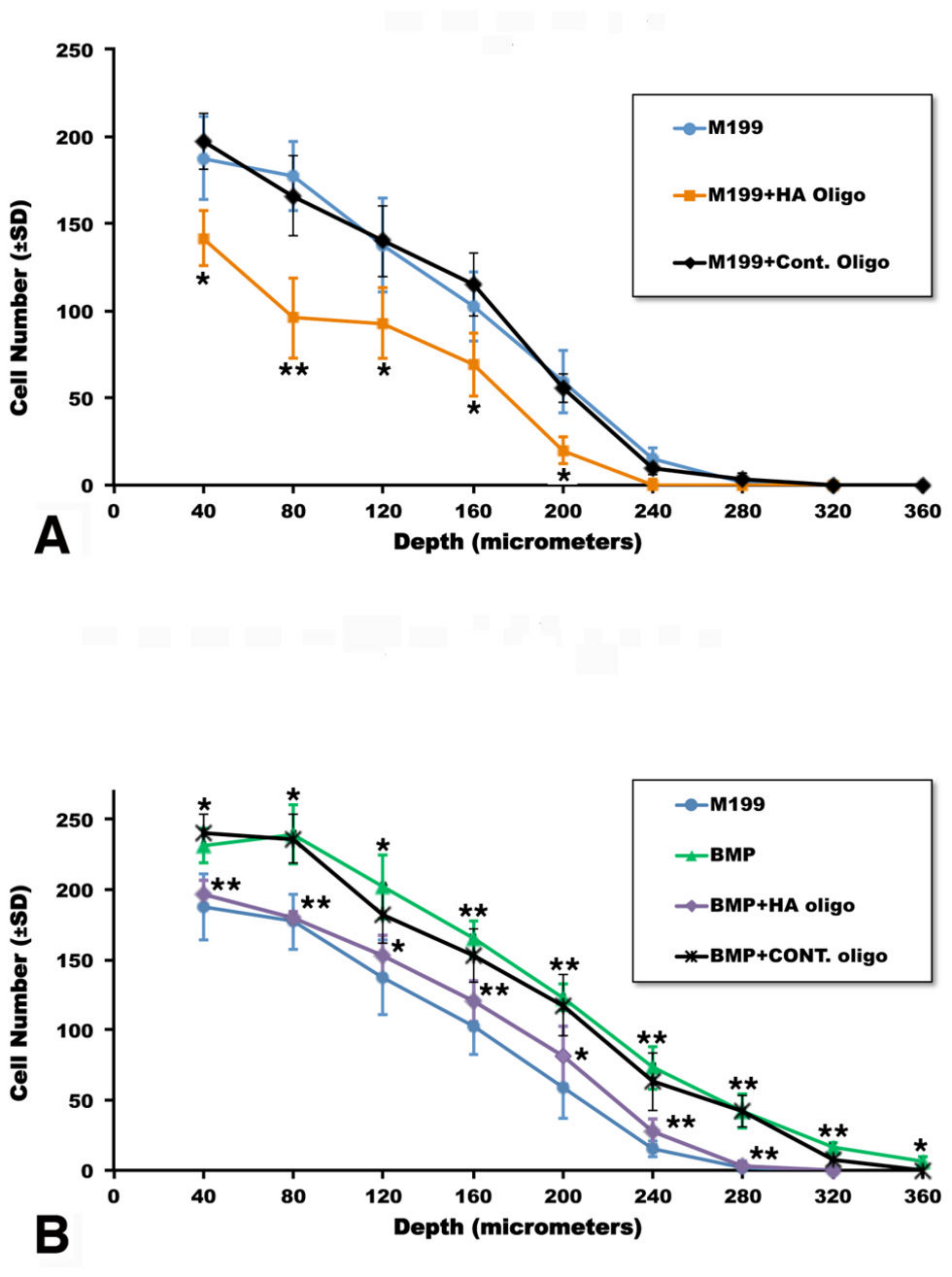
Quantitative evaluation of cell migration by HH stage-24 AV CMC aggregate cultures treated with HA oligomers. (A) CMC aggregate cultures were treated with HA oligomers (100 µg/ml) or chitin oligomers (control oligomers) (100 µg/ml). HA oligomer treatment significantly reduced cell migration, whereas control chitin oligomer treatment did not significantly alter cell migration. *p<0.01; **p<0.001 as compared to the untreated control (M199). (B) BMP-2 (200 ng/ml) promoted cell migration. BMP-2-promoted cell migration was significantly reduced by adding HA oligomers (100 µg/ml) to the CMC aggregate cultures, whereas chitin oligomer treatment did not significantly reduce BMP-2-promoted cell migration. The asterisks on the green curve show significant differences between BMP-2 treatment and the untreated control (M199) (blue curve). The asterisks on the purple curve indicate significant differences between treatment with BMP-2+ HA oligomers and treatment with BMP-2 (green curve). Vertical bars indicate ± SD of the mean. *p<0.01; **p<0.001. A and B represent quantitation of results for each culture condition from a minimum of three experiments, in each of which three separate aggregates were examined.

To examine whether blockade of HA signaling alters BMP-2-induced AV CMC migration, HA oligomers were added to the AV CMC aggregates cultured on the collagen gels. Many studies have shown that the HA oligomers used in this study effectively reduced HA signaling [27] and consequently cell invasion [28,29]. In the present study, we observed that BMP-2-promoted cell migration (Figure 6a, 6b, 6e, 6f) was largely reduced when CMCs were treated with HA oligomers (100 µg/ml) in the presence of BMP-2 (Figure 6c, 6g). HA oligomers alone also repressed migration of CMCs (Figure 6j, 6l) when compared to control chitin oligomer- treated CMCs (Figure 6i, 6k). Chitin oligomers did not alter the effect of BMP-2 stimulation upon cell migration (Figure 6d, 6h). Quantitative analysis confirmed that HA oligomer treatment significantly reduced endogenous and BMP-2-promoted AV cushion cell migration into the collagen gels (Figure 7A, 7B).

Consistent with our previous finding [14], BMP-2 did not induce AV CMC proliferation. Also, treatments with HA oligomers, chitin oligomers, versican siRNA or control (scrambled) RNA did not significantly alter CMC proliferation (Figure S2, S3). Therefore, it is unlikely that the cell migration data we present in this paper reflect altered cell proliferation of CMCs. Moreover, only a few cells (less than 0.1%) underwent apoptosis in the CMC aggregate cultures (Table S1, S2). Therefore, it is reasonable to conclude that the reduced cell numbers found in the collagen gels after treatment with versican siRNA or HA oligomers were due to fewer cells migrating into the gels from the CMC aggregates cultured on the gel surface. 

## Discussion

Studies with *Nkx2*.*5*-cre*/BMP-2* cKO mice showed that myocardially-derived BMP-2 was essential for AV cushion formation [7,8]. Moreover, data from *Tie1*-cre/*Alk3* and *Flk1*-cre/*Alk3* cKO mice indicated a critical role for the Type I BMP receptor, Alk3 in endothelial cells with respect to AV cushion formation [9,10]. Over all, these data revealed a crucial paracrine signaling role for myocardially-derived BMP-2 in AV cushion formation. However, subsequent lethality of the cKO embryos at the initial cushion formation stage has hampered analyzing the role of BMP signaling in later stages of valvulogenesis. Although myocardial expression of BMP-2 is down regulated in the AV myocardium in the later stages, BMP-2 is expressed in AV cushion mesenchyme in mice [5] and chicks [12]. Also, Type I BMP receptors, *Alk3*, *Alk2* and *Alk6* are all expressed in the AV cushion mesenchyme [14], suggesting potential autocrine regulation by BMP-2 in CMCs during post-EMT expansion and maturation of AV cushions. However, the roles of BMP signaling have not been fully understood in CMCs during AV cushion expansion and maturation. Our present work has revealed critical roles for BMP-2 in regulating cell biological processes necessary for distal outgrowth/expansion of AV cushions, valvulogenic ECM production and mesenchymal cell migration by using a well-defined 3D AV CMC aggregate culture system.

It is critical to use a well-defined 3D culture system that recapitulates the *in vivo* microenvironment through which AV cushion cells migrate to access their cellular biological function. An advantage of the 3D AV CMC aggregate culture that we developed is that, because CMCs are dissociated during preparation, only minimal amounts of ECM protein remain upon initiation of the cultures; hence, assessment of BMP-dependent changes in ECM protein expression can be monitored unambiguously. Another advantage is that the original cell number is easily quantified for subsequent monitoring in response to BMP signaling. Thus our culture system satisfies the criteria necessary to assess the role of BMP signaling in AV cushion expansion and maturation. Using this culture system we successfully performed immuno-intensity analysis of ECM component expression, QRT-PCR of ECM components and cell migration assays and found that BMP-2 played significant roles in CMC migration and the production of valvulogenic ECM components by CMCs.

Versican, one of the major ECM components in AV cushions, is a large chondroitin sulfate proteoglycan (CSPG) consisting of a core protein with 12-15 chondroitin sulfate (CS) side chains covalently attached. Versican belongs to a family of extracellular proteoglycans (hyalectins) that bind to HA of which aggrecan, the major proteoglycan of cartilage, is the prototype [30]. The linear glycosaminoglycan, HA, a major constituent of cardiac jelly [31], is synthesized at the plasma membrane by HA synthase (HAS) enzymes HAS1, -2, and -3. *Has2* is the only known HA synthase in the embryonic heart and is essential for AV cushion morphogenesis [22]. *Has2* and *versican* mRNAs were shown to be intensely expressed in the myocardium of the AV canal at the onset of AV cushion formation at ED 9.5 and in AV cushion mesenchyme following EMT at ED10.5 in mice [22]. Versican protein and HA were also localized in mouse AV and OT cushions at ED 9.5 to ED 12.5 [32,33]. In chick, *Has2* mRNA is expressed in the AV canal and OT at the onset of AV cushion formation and restricted to cushion mesenchyme at HH stage-20 [24]. Here we present for the first time that HA and versican protein are intensely expressed in the AV cushion mesenchyme up to the cushion maturation stage (HH stage-29) in the chick. Consistent with semi-quantitative RT-PCR data [26], we found that aggrecan protein expression was less intense in the AV cushion mesenchyme at HH stage-29 than at stage-24. Others found that BMP-2 induced aggrecan expression from AV cushion mesenchymal cells in culture [17], suggesting that BMP-2 can regulate cartilage-associated genes and phenotypes in AV cushions. Our localization data provide the basis for studies to test the hypothesis that BMP signaling in cushion mesenchyme regulates HA and versican during post-EMT AV cushion expansion and maturation. 

A few studies have focused on the regulation of versican and HA/*Has2* expression in cardiac development. TGFbeta2 was found to promote *Has2* expression and HA production during epicardial cell differentiation and invasion [34]. Aberrant accumulation of versican protein was also reported in developing pulmonary valves in ADAMTS5 deficient mice in later stage valvulogenesis [35]. Regarding AV cushion formation, myocardial misexpression of *Tbx2* induced *Has2* expression in the myocardium and accelerated abnormal endocardial cushion formation from the ventricular and atrial myocardium with excessive deposition of HA [36]. Intriguingly, BMP2 was shown to selectively induce *cTbx2* in non-cardiogenic mesoderm in chick embryos [37], suggesting the presence of a BMP--*Tbx2*--*Has2* regulatory axis in cardiac development. Moreover, recent studies revealed that myocardial specific conditional deletion of *Tbx2* and *Tbx3* led to deficiencies in AV cushion formation in mice [38]. Taken together, these findings suggest that *Tbx2* in the AV myocardium regulates *Has2* expression and HA deposition into the cardiac jelly prior to the onset of endocardial EMT and early formation of cushion mesenchyme. However, *Has2* expression was shown to be independent of BMP-2 expression in the precardiac mesoderm [39], suggesting that regulation of HA/*Has2* expression is largely dependent on cell type and developmental context. In this regard, our present work is the first to indicate that BMP-2 induces mRNA expression of *Has2* and *versican* and production of HA and versican protein by CMCs during AV cushion expansion and maturation. It is known that mRNA of versican isoforms, V0, V1 and V2 are expressed in the mouse embryo hearts from ED 10.5-ED 16.5 during development [32]. Because the QRT-PCR primers we used for the present study are not isoform specific but designed to detect all isoforms of chick versican it is of future interest to examine whether BMP-2 induces chick versican mRNA in an isoform-specific manner in post-EMT AV CMCs. In addition, because our mouse developmental data indicate that versican protein expression and HA deposition remain intense in all four cardiac valves at the newborn stage [40], subsequent investigations are needed to determine whether HA and versican play significant roles in later valve leaflet formation and stratification of mature valves. 

Cell migration through ECM after formation of AV cushion mesenchyme is an essential and critical biological process during AV cushion expansion and maturation. HA and versican are known to regulate migration and invasion of many cell types including tumor cells [41,42], hypaxial muscle cells and neural crest cells [43-45]. The role of a primary HA receptor, CD 44 in cell migration and motility has also been extensively studied in tumor cells [46-48] and other cell types [47,49]. HA and versican are prominent ECM components of AV cushions; however, the role of HA and versican in AV CMC migration has received little attention. A mouse model with versican deficiencies (hdf mouse) dies at ED 10.5 and exhibits complete failure in endocardial cushion formation; however, excised AV regions of the hdf mouse have the ability to produce mesenchyme in a 3D collagen gel environment [20]. Intriguingly, *Vcan* ^*∆3/∆3*^ mice whose versican lacks a subdomain of the G1 domain exhibit impaired CMC migration in ED 12.5 AV explant cultures [50]. These data suggest that versican may not be directly involved in CMC formation through an EMT but may regulate CMC migration after EMT in AV cushions. Using a well-defined 3D cell aggregate culture, we demonstrate in the present study that HA production and versican expression contribute to BMP-2-induced CMC migration. Our data also suggest that endogenous HA and versican may play significant roles in CMC migration because cell migration was reduced when AV CMC aggregates were cultured with versican siRNA or HA oligomers in untreated control medium. However, it is still not clear how BMP and/or HA/versican signaling regulate changes in cytoskeletal organization associated with AV endocardial cushion cell migration. HA binding to astrocytes is reported to stimulate Rac1-dependent PKNgammakinase activity, which, in turn, activates the cytoskeletal protein, cortactin and regulates cell migration [51]. Myosin-X was shown to be responsible for BMP-6-dependent filopodial extension and migration of endothelial cells [52]. More recently, actin cytoskeleton reorganization via the p38/MK2/Hsp25 pathway was shown to be critical for BMP-2-induced migration of C2C12 myoblasts [53]. It is of future interest to determine how BMP-2 and /or BMP2-induced HA/versican regulates cytoskeletal organization and filopodial extension of CMCs during post-EMT AV cushion expansion and maturation.

In conclusion, our current work provides evidence that BMP-2 induces expression and production of versican and HA by AV CMCs and that versican and HA contribute to BMP2-induced CMC migration by using a well-defined 3D CMC aggregate culture. BMP-2 also induces other valvulogenic molecules including an ECM protein, periostin [14] which is reported to regulate cellular migration, collagen fibrillogenesis and compaction in post-EMT AV cushion maturation [54]. Because many other molecules are expressed in the post-EMT cushion mesenchyme and involved in valvulogenesis [2-4,24], further studies are warranted to better characterize the intersection of BMP signaling with other valvulogenic regulatory molecules and their regulatory cascades in post-EMT AV valvulogenesis. 

## Materials and Methods

### Chick embryos

Fertilized eggs of White Leghorn (*Gallus gallus domesticus*) chicken were purchased from Pilgrim’s Pride (Sumter, SC) and incubated in a humid atmosphere at 37°C. Stages of embryonic development were determined by using the criteria of Hamburger and Hamilton (HH) [55].

### Histochemical Localization of Versican, Aggrecan and Hyaluronan (HA)

Preparation and specification of antibodies to anti-chick versican and aggrecan were previously reported [26]. The anti-chick versican antibody used in the present study was prepared against a synthetic peptide whose sequence was found in the alternatively spliced GAG-alpha region of versican and should recognize the V0 and V2 forms of versican but should not recognize the V1 form [26]. HA deposition was histochemically detected by using a specific hyaluronan binding protein (HABP) following the procedure previously described [25]. Biotinylated HABP (bHABP) was purchased from Seikagaku America (East Falmouth, MA). 

Chick embryos from HH stage-17 to stage-29 were fixed in 100% methanol at -20°C overnight and processed for paraffin embedding as described previously [56]. For immunohistochemical localization of versican and aggrecan, deparaffinized 6-µm sections were treated with 10% normal goat serum (NGS) in 1% bovine serum albumin (BSA) in phosphate buffered saline (PBS) to block non-specific binding and incubated with either anti-chick versican (final concentration, 2 µg/ml) or anti-chick aggrecan (final concentration, 2 µg/ml) in 1%BSA/PBS. After rinsing, 10 µg/ml RITC–labeled goat anti-rabbit IgG (ICN Pharmaceuticals, Aurora, OH) was applied. After versican or aggrecan immunostaining, MF20 (anti-chick sarcomeric myosin) immunostaining was performed with10 µg/ml Cy5-labbeled donkey anti-mouse IgG (Jackson Laboratories, West Grove, PA). For histochemical localization of HA, deparaffinized sections were incubated with bHABP in 10% NGS/1%BSA/PBS. After rinsing, sections were incubated with FITC-conjugated streptavidin (1:100, Amersham, Littlechalfont Buckinghamshire, UK). Stained sections were examined under Leica TCS Sp5 microscope. 

### 3D AV endocardial cushion mesenchymal cell (CMC) aggregate culture

3D AV CMC aggregate culture was performed as described previously [14] with minor modifications. An advantage of our 3D aggregate culture is that mesenchymal cells are dissociated with trypsinization before the hanging-drop procedure, therefore cells are carrying minimal amount of endogenous extracellular matrix (ECM) proteins that can hamper the assessment of ECM production. Cushion mesenchyme was dissected out from HH stage-24 chick AV cushions by carefully removing the associated myocardium. AV CMCs were dissociated with trypsinization and 40,000 CMCs were cultured as a hanging drop overnight with serum-free Medium199 (Invitrogen) supplemented with ITS (5 µg/ml insulin, 5 µg/ml transferring, 5 ng/ml selenium, BD Sciences) and antibiotics (100 units/ml penicillin and 100µg/ml streptomycin, Invitrogen). Resultant cell aggregates were placed on the hydrated collagen gels (1mg/ml, rat tail tendon, BD Biosciences, Bedford, MA) and cultured with Medium 199 supplemented with 1% chick serum (Sigma, St. Louis, MO), ITS and antibiotics. Recombinant human BMP-2 and Noggin were purchased from R&D Systems (Minneapolis, MN). BMP-2 was used at final concentration ranging from 20 to 200 ng/ml and Noggin was used at final concentration of 500 ng/ml. Cultures were observed daily under an inverted microscope with Hoffman optics (Olympus, IMT-2). Cell migration, expression of versican and HA deposition, and mRNA expression of *versican* and *Has2* were evaluated at 72 hrs. For each AV CMC culture experiment, three CMC aggregates were examined for each culture condition and the experiments were repeated a minimum of three times.

### Cell migration assay

Seventy-two hours after placing the AV CMC aggregates on collagen gels, cell migration was assessed under an inverted microscope with Hoffman optics (Olympus, IMT-2). Cell numbers were counted in a series of focal planes at 40µm intervals within the collagen gels from 40µm below the surface of the gels to the bottom. Quantitative analysis data for cell migration assay represent a minimum of three independent experiments with cultures performed in triplicate.

### BrdU incorporation assay

BrdU incorporation was used as an index of cell proliferation. 5’-bromodeoxyuridine (BrdU, Sigma) was added to a final concentration of 50 µM for 2 hrs before the termination of the CMC culture. CMC aggregates cultured on the collagen gels were fixed with 4% paraformaldehyde in PBS, pH 7.4 and rinsed with PBS. CMC aggregates were permeabilized with 0.1% Triton X-100/PBS and treated with DNase I (0.1 U/µl; Roche Diagnostics). Then, CMC aggregates were washed, blocked with 3% BSA/0.1% TritonX-100/PBS and processed for immunohistochemical detection of BrdU in the nuclei with anti-BrdU (BD Sciences) as described previously [14]. All nuclei were stained with propidium iodide. Stained samples were observed under a Leica TCS SP5 confocal microscope. Pictures for the BrdU incorporation assay were taken from the middle of the entire thickness of the CMC aggregates. A total of 500 nuclei was evaluated on photographs taken from 5 random fields per each CMC aggregate culture. Quantitative analysis data for BrdU incorporation assay represent a minimum of three independent experiments with cultures performed in triplicate.

### TUNEL assay

Terminal deoxynucleotidyl transferase dUTP nick end labeling (TUNEL) assay was performed to detect and quantify apoptosis at single cell level using the In Situ Cell Death Detection Kit (Roche). Following manufacturer’s recommendation, CMC aggregates cultured on collagen coated wells were fixed with 4% paraformaldehyde in PBS and after being rinsed with PBS and permeabilization, CMC aggregates were processed for TUNEL reaction. All nuclei were stained with propidium iodide. TUNEL positive cells in CMC aggregates were observed and counted under a Leica TCS SP5 confocal microscope. Quantitative analysis data for TUNEL assay represent a minimum of three independent experiments with cultures performed in triplicate.

### Immunostaining, histochemical detection and staining intensity analysis of AV CMC aggregate culture

Immunostaining and staining intensity analysis for CMC aggregates cultured on collagen gels were performed as described before [14] with minor modifications. Briefly, cultured CMC aggregates were fixed in 100% methanol at -20 °C and processed for immunostaining with anti-chick versican and histochemical detection of HA using bHABP. Stained samples were observed under a confocal microscope (Leica TCS SP5). The staining intensity of versican and HABP was evaluated by measuring the intensity of the fluorescence of the cell aggregates on photographs using computer software, Adobe Photoshop CS2. Results of staining intensity analysis represent at least three independent experiments with cultures performed in triplicate.

### Quantitative RT-PCR for versican and Has2

Total RNA was extracted and purified using RNA STAT-60 (Tel-Test, Inc., Friendswood, TX) from CMC aggregates that had been cultured for 72 hrs on the collagen gels. After DNase digestion to remove genomic DNA, mRNA was further purified by Arcturus PicoPure RNA Isolation kit (AB applied biosystems, Foster City, CA). Complementary DNA was prepared using the iScript^TM^ cDNA synthesis kit (BIO RAD, Hercules, CA) following the manufacturer’s instruction. The following PCR primer pairs were designed to specifically amplify chick *versican* (*NM_204787*) (Forward 5’-aacatatggacgccgttttc-3’; Reverse 5’-cattcctccaggccacatac-3’) and chick *Has2* (AF106940) (Forward, 5’-ccctgaaaaagtgcgatttg-3’; Reverse 3’-gcagtcctttggtcatagagg-5’). Versican primer sequences used in this study are located in the versican G1 domain and detecting all isoforms of chick versican. Real-time PCR reactions were performed using 40 cycles of 94°C for 30 sec, 56°C for 30 sec and 72°C for 2 min. Chick *versican* and *Has2* amplification was normalized to chick ß-actin amplification for each experimental treatment. The PCR products were verified via the thermal cycle sequencing using TagDNA polymerase and fluorescent dye-labeled termination. (MUSC, Biotechnology Resources Laboratory). QRT-PCR results represent at least three independent experiments with cultures performed in triplicate. 

### HA oligomer treatment

HA oligomers were fractionated from testicular hyaluronidase digests of purified hyaluronan polymer by differential filtration as described previously [57]. The oligomers used in this study were a mixed fraction of average molecular weight ~2.5 X 10^3^ and were composed of 3-10 disaccharide units. The specificity of HA oligomers was analyzed as described before [27]. Chitin oligomers, used as control to HA oligomers, were obtained from Seikagaku America (Falmouth, MA). CMC aggregates were treated with 100 µg/ml HA or chitin oligomers in serum-free Medium 199 for 4 hrs and cultured in Medium 199 supplemented with 1% chick serum and ITS as indicated above. 

### Versican siRNA treatment

Nucleotide RNA duplexes corresponding to the chick *versican* mRNA (*NM_204787*) were designed using BLOCK-iT^TM^ RNAi Designer (Invitrogen, Carlsbad, CA) and generated by Invitrogen (Carsbad, CA). The chick versican siRNA oligonucleotides used for this study were stealth103, targeting chick versican mRNA 333-357 and stealth 208, targeting chick versican mRNA 438-462. A scrambled (control) oligonucleotide (sense 5’-CAAGGCUAAGAUGGUAAUCGUAGAU-3’ antisense 5’- AUCUACGAUUACCAUCUUAGCCUUG-3’) was also generated by Invitrogen. Target sequences are located in the chick versican G1 domain which contains HA-binding sites and targeting all chick versican isoforms. Two siRNAs were combined and a final siRNA concentration of 100 nM was used with Lipofectamine 2000 (Invitrogen) with serum-free Medium 199 for 4 hrs to transfect CMC aggregates following the manufacturer’s recommendation. A scrambled (control) oligonucleotide was also used at a final concentration of 100 nM with Lipofectamine 2000. To measure the transfection efficiency, the BLOCK-iT^TM^ Fluorescent (FL) Oligo (Invitrogen) was co-transfected. More than 75% of CMCs on average showed FL oligo transfection (not shown). After a 72 hrs culture with 1% chick serum and ITS as described above, the reduction of versican protein and mRNA expression by versican siRNA transfection with CMCs was confirmed by real time RT-PCR and versican immunointensity analysis (Figure S1). 

### Statistical analyses

Analysis of variance (ANOVA) was used to determine significant differences between groups. When differences were found among groups, the Turkey-Kramer’s t-test was used to determine if the difference was statistically significant. Differences were considered significant if *p<0.05*. 

## Supporting Information

Figure S1 **Versican expression in siRNA-treated AV CMC aggregate cultures from HH stage-24 chick embryos.** (A-C) Versican immunostaining in AV endocardial CMC aggregate culture. The CMC aggregates were cultured with Medium 199 alone (untreated control) (A), M199+versican siRNA (100 nM) (B) or M199+ control (scrambled) RNA (100 nM) (C). Versican siRNA treatments significantly reduced versican immunostaining (B), whereas control (scrambled) RNA did not reduce immunostaining (C). (D) Quantitative evaluation of versican immunointensity in AV CMC aggregate culture. Versican immunointensity was significantly reduced in versican siRNA-treated culture, whereas scrambled RNA-treated culture did not show significant reduction of versican immunointensity. (E) QRT-PCR data revealed an approximately 80% reduction of versican mRNA in CMC aggregate culture treated with versican siRNA, whereas scrambled RNA treatments did not significantly reduce versican mRNA expression. Vertical bars indicate ± SD of the mean. *p<0.05, as compared to the untreated control (M199).
(PSD)

Figure S2 **BrdU incorporation assay for HA oligomer treatment.** The CMC aggregates were cultured in untreated control (M199) (A, D), or treated with HA oligomers (100 µg/ml) (B, E) or chitin oligomers (control oligomers) (100 µg/ml) (C, F) in the presence (D, E, F) or absence (A, B, C) of BMP-2 (200 ng/ml). All nuclei were stained with propidium iodide (red). Note that incidence of BrdU-positive (green) nuclei appears to be the same in all CMC cultures. (G) Quantitative analysis of BrdU incorporation assay with AV CMCs. BrdU-positive and –negative nuclei in the cultured CMCs were counted to determine the percentage of cells in cell-cycle transit. A total of 500 nuclei in each culture was evaluated in 5 random fields. Vertical bars indicate ± SD of the mean. There were no significant differences between treated (BMP-2, HA oligomer, and chitin oligomer) and untreated control (M199) cultures.
(TIF)

Figure S3 **BrdU incorporation assay for versican siRNA treatment.** The CMC aggregates were incubated in untreated control (M199) (A, D), or treated with versican siRNA (100 nM) (B, E) or a scrambled (control) RNA (100 nM) (C, F) in the presence (D, E, F) or absence (A, B, C) of BMP-2 (200 ng/ml). All nuclei were stained with propidium iodide (red). Note that incidence of BrdU-positive (green) nuclei appears to be the same in all CMC cultures. (G) Quantitative analysis of BrdU incorporation assay with AV CMCs. BrdU-positive and –negative nuclei in the cultured CMCs were counted to determine the percentage of cells in cell-cycle transit. A total of 500 nuclei in each culture were evaluated in 5 random fields. Vertical bars indicate ± SD of the mean. There were no significant differences between treated (BMP-2, versican siRNA, and scrambled RNA) and untreated control (M199) cultures.
(TIF)

Table S1 **TUNEL assay for versican siRNA treatment.** CMC aggregates of 40,000 cells were untreated (M199), or treated with versican siRNA (100 nM) or scrambled RNA (100 nM) in the presence or absence of BMP-2 (200 ng/ml). Only a few TUNEL positive cells (22-36 cells/aggregate) were found in CMC cultures. Values are expressed as percentage of total cell number.
(DOC)

Table S2 **TUNEL assay for HA oligomer treatment.** CMC aggregates of 40,000 cells were untreated (M199), or treated with HA oligomers (100 µg/ml) or chitin oligomers (control oligomers) (100 µg/ml) in the presence or absence of BMP-2 (200 ng/ml). Only a few TUNEL positive cells (22-26 cells/aggregate) were found in CMC cultures. Values are expressed as percentage of total cell number.
(DOC)
