# Supplementary figures and images for: BMP-2 Induces Versican and Hyaluronan That Contribute to Post-EMT AV Cushion Cell Migration
Source: PLoS One. 2013 Oct 11;8(10):e77593. doi: 10.1371/journal.pone.0077593 (PMC3795687; doi:10.1371/journal.pone.0077593)

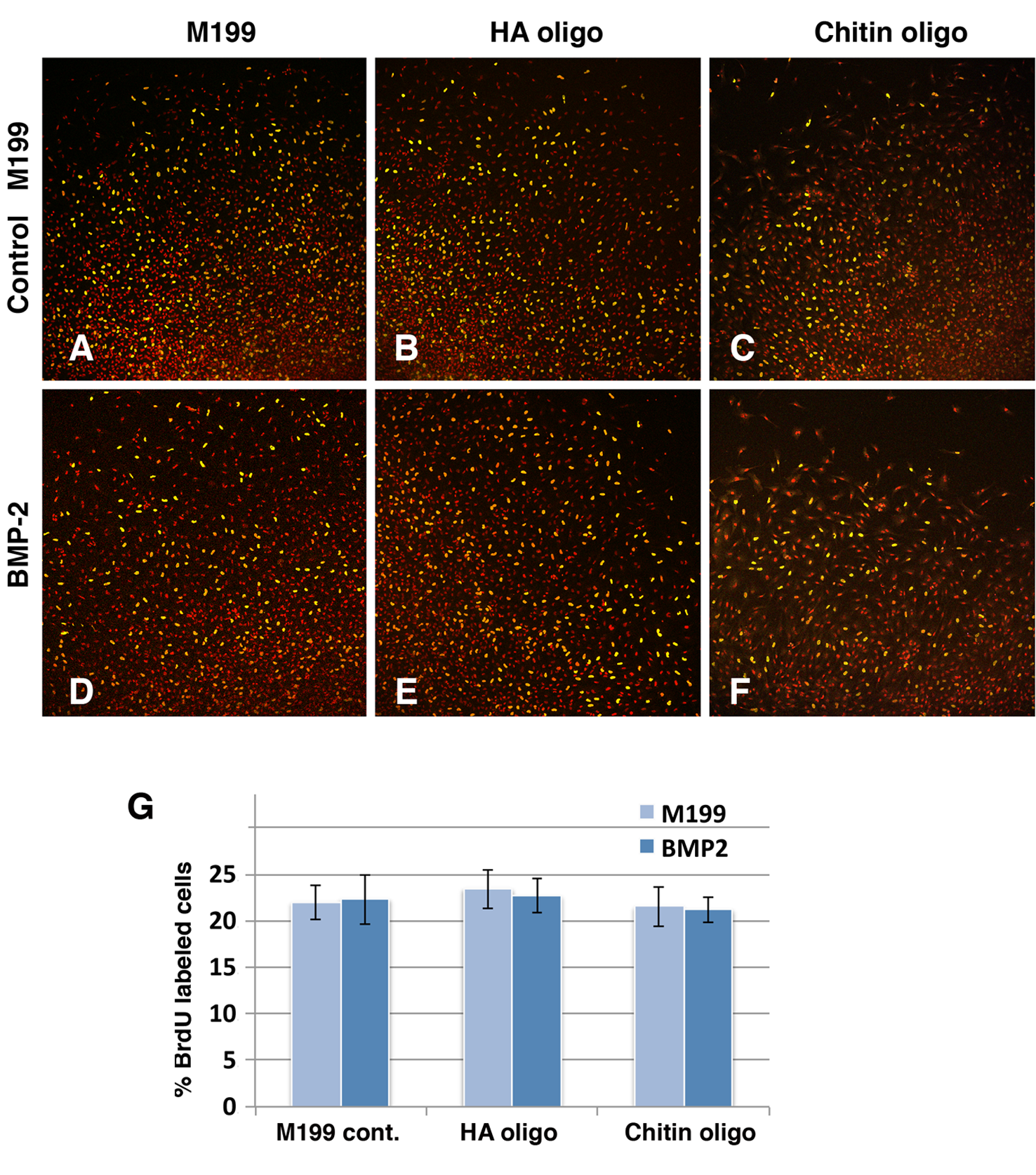

Supplement: Figure S2 — BrdU incorporation assay for HA oligomer treatment. The CMC aggregates were cultured in untreated control (M199) (A, D), or treated with HA oligomers (100 µg/ml) (B, E) or chitin oligomers (control oligomers) (100 µg/ml) (C, F) in the presence (D, E, F) or absence (A, B, C) of BMP-2 (200 ng/ml). All nuclei were stained with propidium iodide (red). Note that incidence of BrdU-positive (green) nuclei appears to be the same in all CMC cultures. (G) Quantitative analysis of BrdU incorporation assay with AV CMCs. BrdU-positive and –negative nuclei in the cultured CMCs were counted to determine the percentage of cells in cell-cycle transit. A total of 500 nuclei in each culture was evaluated in 5 random fields. Vertical bars indicate ± SD of the mean. There were no significant differences between treated (BMP-2, HA oligomer, and chitin oligomer) and untreated control (M199) cultures. (TIF) [file pone.0077593.s002.tif]

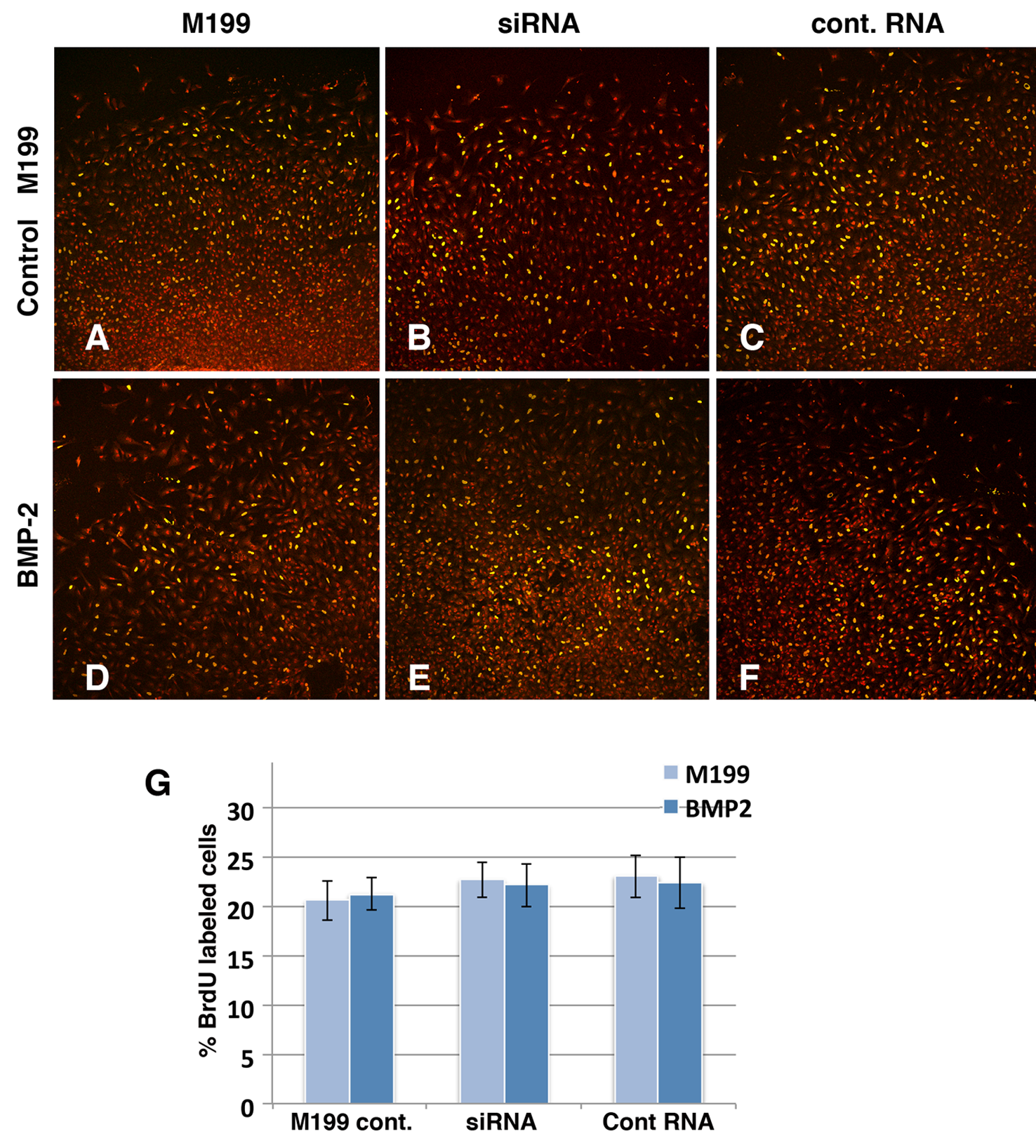

Supplement: Figure S3 — BrdU incorporation assay for versican siRNA treatment. The CMC aggregates were incubated in untreated control (M199) (A, D), or treated with versican siRNA (100 nM) (B, E) or a scrambled (control) RNA (100 nM) (C, F) in the presence (D, E, F) or absence (A, B, C) of BMP-2 (200 ng/ml). All nuclei were stained with propidium iodide (red). Note that incidence of BrdU-positive (green) nuclei appears to be the same in all CMC cultures. (G) Quantitative analysis of BrdU incorporation assay with AV CMCs. BrdU-positive and –negative nuclei in the cultured CMCs were counted to determine the percentage of cells in cell-cycle transit. A total of 500 nuclei in each culture were evaluated in 5 random fields. Vertical bars indicate ± SD of the mean. There were no significant differences between treated (BMP-2, versican siRNA, and scrambled RNA) and untreated control (M199) cultures. (TIF) [file pone.0077593.s003.tif]
