# Supplementary material for: BMP-2 Induces Versican and Hyaluronan That Contribute to Post-EMT AV Cushion Cell Migration
Source: PLoS One. 2013 Oct 11;8(10):e77593. doi: 10.1371/journal.pone.0077593 (PMC3795687; doi:10.1371/journal.pone.0077593)
Supplement: Table S1 — TUNEL assay for versican siRNA treatment. CMC aggregates of 40,000 cells were untreated (M199), or treated with versican siRNA (100 nM) or scrambled RNA (100 nM) in the presence or absence of BMP-2 (200 ng/ml). Only a few TUNEL positive cells (22-36 cells/aggregate) were found in CMC cultures. Values are expressed as percentage of total cell number. (DOC) [file pone.0077593.s004.doc]

**Table S1. TUNEL assay for versican siRNA treatment***

|  | **M199** | **siRNA** | **Cont RNA** |
| --- | --- | --- | --- |
| **M199** | 0.057 ± 0.015 | 0.086 ± 0.013 | 0.075 ± 0.017 |
| **BMP2** | 0.057 ± 0.015 | 0.089 ± 0.018 | 0.074 ± 0.015 |

***** Values are expressed as percentage of total cell number.
