# Supplementary material for: BMP-2 Induces Versican and Hyaluronan That Contribute to Post-EMT AV Cushion Cell Migration
Source: PLoS One. 2013 Oct 11;8(10):e77593. doi: 10.1371/journal.pone.0077593 (PMC3795687; doi:10.1371/journal.pone.0077593)
Supplement: Table S2 — TUNEL assay for HA oligomer treatment. CMC aggregates of 40,000 cells were untreated (M199), or treated with HA oligomers (100 µg/ml) or chitin oligomers (control oligomers) (100 µg/ml) in the presence or absence of BMP-2 (200 ng/ml). Only a few TUNEL positive cells (22-26 cells/aggregate) were found in CMC cultures. Values are expressed as percentage of total cell number. (DOC) [file pone.0077593.s005.doc]

**Table S2. TUNEL assay for HA oligomer treatment***

|  | **M199** | **HA oligo** | **Chitin oligo** |
| --- | --- | --- | --- |
| **M199** | 0.056 ± 0.006 | 0.067 ± 0.006 | 0.065 ± 0.016 |
| **BMP2** | 0.063 ± 0.016 | 0.059 ± 0.002 | 0.060 ± 0.008 |

***** Values are expressed as percentage of total cell number.
